# Exploration of the Content and Structure of Preferences for Leisure Activities of People Receiving Adult Day Services Using Concept Mapping

**DOI:** 10.1093/geront/gnad142

**Published:** 2023-10-23

**Authors:** Mike Rommerskirch-Manietta, Johannes M Bergmann, Christina Manietta, Daniel Purwins, Kimberly Van Haitsma, Katherine M Abbott, Martina Roes

**Affiliations:** Deutsches Zentrum für Neurodegenerative Erkrankungen (DZNE), Witten, North Rhine-Westphalia, Germany; Faculty of Health, School of Nursing Science, Witten/Herdecke University, Witten, North Rhine-Westphalia, Germany; Deutsches Zentrum für Neurodegenerative Erkrankungen (DZNE), Witten, North Rhine-Westphalia, Germany; Faculty of Health, School of Nursing Science, Witten/Herdecke University, Witten, North Rhine-Westphalia, Germany; Deutsches Zentrum für Neurodegenerative Erkrankungen (DZNE), Witten, North Rhine-Westphalia, Germany; Faculty of Health, School of Nursing Science, Witten/Herdecke University, Witten, North Rhine-Westphalia, Germany; Diakonie Osnabrück, Stadt und Land GmbH, Osnabrück, Lower Saxony, Germany; College of Nursing, Pennsylvania State University, University Park, Pennsylvania, USA; Department of Sociology and Gerontology, Miami University, Oxford, Ohio, USA; Scripps Gerontology Center, Miami University, Oxford, Ohio, USA; Deutsches Zentrum für Neurodegenerative Erkrankungen (DZNE), Witten, North Rhine-Westphalia, Germany; Faculty of Health, School of Nursing Science, Witten/Herdecke University, Witten, North Rhine-Westphalia, Germany

**Keywords:** Community, Dementia, Long-term care, Person-centered, Public involvement

## Abstract

**Background and Objectives:**

Providing preferred leisure activities appears to be an important approach to support and empower people receiving adult day services (ADS) allowing them to age in place. To provide the conceptualization for a preference instrument, we actively involved people receiving ADS in exploring the content and structure of their preferences for leisure activities.

**Research Design and Methods:**

We chose a concept mapping methodology and involved 16 people receiving ADS. We systematically reviewed the literature and conducted semistructured interviews to *generate* a set of 80 preferences. Analysis of *structuring* these preferences resulted in a 3-dimensional cube with 12 clusters. A graphical representation was then *interpreted*, and the clusters were labeled.

**Results:**

Our conceptualization divides preferences for leisure activities into the following: 1. *Take a trip*, 2. *Revel in memories and catch up on the news* (most important), 3. *Do something for yourself and come to rest*, 4. *Play intelligence and parlor games*, 5. *Make/produce and try something alone or in a group*, 6. *Keep fit and cheer others on in sports* (least important), 7. *Learn, educate, and share knowledge*, 8. *Have contact with other people*, 9. *Attend at entertainment, cultural, and amusement events*, 10. *Enjoy music, your homeland, or other countries*, 11. *Engage in outdoor activities*, and 12. *Get involved, offer support, and provide companionship*.

**Discussion and Implications:**

Our results may lead to the development of instruments and thus opens the field for further research and theory building on preferences for leisure activities of people receiving ADS.

## Background

Participation of older people in leisure activities is a prerequisite for healthy aging. Leisure activities offer the opportunity to maintain and promote physical and mental health, social engagement, and prevent diseases (Bone et al., 2022; Watts et al., 2022; Wion et al., 2022), which are also key aspects to achieve successful aging (Rowe & Kahn, 1997). According to self-determination theory, activities of interest (e.g., leisure activities) provide the possibility to address and satisfy one’s psychological needs for competence, relatedness, and autonomy, thus promoting personal growth that leads to well-being (Deci & Ryan, 2000; Van Haitsma et al., 2020).

Despite these positive outcomes, participation in leisure activities may decline in older age (Lee et al., 2018). This can be explained by life transitions, for example, losing a partner, requiring care, or moving into a nursing home, which can influence individual leisure behavior (Nimrod & Janke, 2012). Accordingly, these life transitions can result in various barriers (e.g., feelings of loneliness, decline in physical and cognitive functioning, and a lack of offers for support) to leisure activities, resulting in compensatory challenges that impede or even prevent further participation (Cohen-Mansfield & Jensen, 2022; Lee et al., 2017; Stoddart et al., 2022; Tak et al., 2015).

Focusing on requiring care in older age as a possible life transition, for example, due to symptoms of dementia, home- and community-based services such as adult day services (ADS) provide support for older people to age in place (Sadarangani, Chong, et al., 2021), which satisfies their preference to avoid moving into a nursing home (Bishop & Degenholtz, 2022). To facilitate this, ADS centers offer professional healthcare services (e.g., medication management) and activities (e.g., bingo) that are *expected* to maintain and/or promote the health and well-being of people receiving ADS and those close to them (e.g., relatives, friends) (Liu et al., 2022; Orellana et al., 2020). Current research in the ADS environment has focused on standardized measurements for ADS key outcomes, person-centered care approaches, and the development and testing of study designs and interventions to investigate the effectiveness of ADS for clients and their caregivers (Gitlin et al., 2023; Roth et al., 2020; Sadarangani, Chong, et al., 2021; Sadarangani, Zagorski, et al., 2021).

To clarify the fuzzy notion of “activities” in the context of ADS (Li et al., 2022; Sadarangani et al., 2022), it seems important to consider that people receiving ADS understand the life transition of receiving ADS as a transition in their place of leisure. This means that people who receive ADS report a shift in their place of leisure from the private domain to the communal ADS environment. This transition is linked to the requirement that ADS providers know about people’s individual leisure activity preferences to creatively address the barriers they face (e.g., decline in physical functioning, stigma, and self-ageism) (Harrison & Ogden, 2019; Jenkin et al., 2021) and to satisfy their preferences with preference-based leisure activity programs (Rommerskirch-Manietta, Purwins, et al., 2023).

Currently, it seems that preferences are rarely considered when planning activity services. For example, older people perceive that activities in ADS centers do not reflect their preferences in activity planning (Orellana et al., 2020). The National Adult Day Services Association reports that activities provided in ADS centers are based on the needs and competencies of people receiving ADS but does not mention preference-based activity services (NADSA, 2023).

Consequently, reframing activities as leisure activities, addressing the requirements described above, and incorporating these requirements into the services provided by ADS centers, offers people receiving ADS the opportunity to maintain participation in their preferred leisure activities. This could lead to an outcome of stable or improved health and well-being and could prevent diseases, which in turn could stabilize or improve care needs (Doroszkiewicz & Sierakowska, 2021). Furthermore, a preference-based living approach, as an operationalization of a person-centered philosophy of care, allows for trusting relationships to build between the people receiving care and care teams (Fazio et al., 2018; Van Haitsma et al., 2020). The realization of a person-centered philosophy of care is accompanied by a positive perception of quality and satisfaction by the people who receive these services (Bucy et al., 2023).

### Prerequisites for a Preference-Based Leisure Activity Program

Lessons learned from a statewide pay-for-performance initiative for the implementation of a preference instrument to enhance person-centered care in the long-term care environment suggested that an important aspect for the provider is the provision of a feasible (e.g., less time-consuming) instrument to systematically assess people’s preferences (Abbott et al., 2021).

There are a variety of different instruments (e.g., Preferences for Everyday Living Inventory) for different environments (e.g., nursing homes) that assess either a broad (more than one topic, for example, assessing preferences for care, leisure, and social contact) or a specific (one topic, e.g., assessing preferences for food) scope of preferences. Instruments with a specific scope can be expected to be less time-consuming than those with a broad scope, as the number of items or questions to ask is usually smaller. However, no instrument currently focuses on the systematic and comprehensive assessment of preferences for leisure activities of people receiving ADS (Rommerskirch-Manietta et al., 2022).

In light of this and in addition to the provider’s perspective of obtaining a feasible instrument, it is prudent to consider the “voice” of the people the instrument is intended to support (e.g., people receiving ADS) when developing an instrument. Considering their “voice” implies developing an instrument “by” and/or “with” them and not “for” them (NIHR, 2021). This “involving” approach is important to understand what items a concept (e.g., preferences for leisure activities) encompasses, from individuals’ life experiences and how they structure them. These points seem to be essential prerequisites for addressing the conceptual complexity of the concept of preferences for leisure activities of people receiving ADS and developing an accurate instrument for assessing this concept (Boateng et al., 2018; Rosas & Ridings, 2017).

### Research Question

To provide the conceptual basis for the development of an instrument to assess preferences for leisure activities, we investigated the following primary research question: “What are the content and structure of preferences for leisure activities of people receiving ADS?”

## Method

### Study Design

To answer our research question, we chose a concept mapping methodology (Trochim & McLinden, 2017). Concept mapping is described as a mixed method that relies on an involving and inductive research approach; this method has been proven effective in instrument development (Rosas & Ridings, 2017). Our concept mapping was part of a larger study (2020–2023) conducted in Germany with the aim of developing and psychometrically testing an instrument for assessing the preferences for leisure activities of people receiving ADS (Rommerskirch-Manietta et al., 2021).

We chose the method of concept mapping for our study because it enabled us to explore the concept of leisure activities of people receiving ADS together with them based on their different life experiences. Furthermore, the various steps of concept mapping gave us the flexibility to adapt the process to the support needs of the people involved, and thus guided us through the research process in a straightforward manner together with the people involved.

Ethical approval was granted by the ethics committee of Witten/Herdecke University (No. 226/2020). Whenever applicable, we follow the Mixed Methods Article Reporting Standards in this article to ensure rigorous and transparent reporting of our concept mapping. For example, we report why concept mapping is a useful method for our study (Levitt et al., 2018).

### Concept Mapping Process

In the following sections, we describe the first five steps (*preparation*, *generation*, *structuring*, *representation*, and *interpretation*) of our concept mapping. The *utilization* of our results as the last step of our concept mapping is explored in our Discussion section (Trochim & McLinden, 2017).

#### Step 1. Preparation

The *preparation* step consists of selecting and recruiting the people to be involved in the concept mapping (Trochim & McLinden, 2017).

We considered people receiving ADS for the involvement in the concept mapping if they (a) used the ADS at least once per week or four times per month, (b) were able to verbally report on their preferences for leisure activities, and (c) were able to sort cards by hand or verbally instruct others to do so on their behalf.

To inform participants about the possibility of being involved in our concept mapping, we contacted our practice partners from a former study (Stacke et al., 2020) and informed them about the upcoming study via e-mail. As a result, three ADS centers agreed to partner with us, helped us disseminate the information in their centers, and contacted people receiving ADS who might be interested in being involved. Two researchers (M. Rommerskirch-Manietta, M. Roes) conducted kick-off meetings with the nursing staff of these ADS centers via Zoom and informed them about the objectives of the study, the inclusion and exclusion criteria, and the focus on a purposive involvement strategy (Patton, 2014). Informational flyers and posters were then provided to the ADS centers, and a researcher (M. Rommerskirch-Manietta) conducted kick-off meetings at the centers to inform interested people receiving ADS about the study, answer their questions, and discuss the study objective with them.

We conducted an ongoing consent approach for the involvement of people receiving ADS in our concept mapping (Rommerskirch-Manietta et al., 2021). A nurse on site at the ADS centers obtained the initial written consent from interested people directly and/or from their legal guardian and forwarded this information to a researcher (M. Rommerskirch-Manietta) via e-mail. Furthermore, this researcher, who has a professional background as a geriatric nurse and experience in working with older people with and without dementia, evaluated the consent for each concept mapping step with the people involved.

As a result, 16 people receiving ADS from three different ADS centers voiced their interest in being involved in our concept mapping and gave their initial written informed consent. Most of the people stated that they were female (*n* = 12) and their ages ranged from 62 to 92 years. Dementia diagnosis was present in most of the people (*n* = 11; Table 1).

**Table 1.** Characteristics of the Involved People

Name^a^	Age	Gender	Origin	Education	Former work	Degree of care needed^b^	Dementia diagnosis (or other cognitive diseases)	Tenure receiving ADS	ADS center	Days per week receiving ADS	Completed the concept mapping step(s)
Ilse	74	Female	Germany	Ninth-grade secondary school	Industrial clerk	5	Yes	2 years	1	4	2
Ludwig	89	Male	Germany	Eighth-grade secondary school	Car mechanic	3	Yes	4 years	2	3	2
Adelia	75	Female	Portugal	Fourth-grade primary school	Post office worker	4	No	4 months	1	2	2
Wolfgang	64	Male	Germany	College; master’s degree	Architect	4	Yes	5 years	3	4	2, 3, & 5
Heinrich	85	Male	Upper Silesia	Seventh-grade secondary school	Welder	4	Yes	1.5 years	2	4	2 & 3
Stenzel	79	Male	Silesia	Basic primary/secondary school	Industrial mechanic	3	Yes	1 month	3	3	2, 3, & 5
Annette	65	Female	Germany	Basic primary/secondary school	Saleswoman	4	(Brain aneurysm)	9 months	1	1	2, 3, & 5
Rita	92	Female	Germany	Basic primary/secondary school	Nurse	2	Yes	11 months	3	3	2, 3, & 5
Jutta	69	Female	Germany	Basic primary/secondary school	Housekeeper	3	(Intellectual disability)	6 months	3	3	2 & 3
Regina	84	Female	Germany	Basic primary/secondary school	Hairstylist	2	Yes	2 months	3	2	2, 3, & 5
Elsbeth	81	Female	Germany	No information	School office worker	3	Yes	3 months	2	2	2
Käthe	85	Female	Germany	2-year business school	Secretary	2	Yes	4 months	3	3	2
Klaus	79	Male	Germany	Basic primary/secondary school	Locksmith	3	Yes	2 years	3	4	2, 3, & 5
Edith	80	Female	Germany	High school	Secretary	3	(Depression, Mania)	2 years	3	3	2, 3, & 5
Sieglinde	85	Female	Germany	Basic primary/secondary school	Housekeeper	3	Yes	5.5 years	3	3	2
Ruth	87	Female	Germany	Basic primary/secondary school	Dry cleaner	4	No	10 years	2	3	3

*Notes*: ADS = adult day services.

^a^Pseudonymized.

^b^Ranges from 0 to 5; higher scores indicate higher dependency on care.

#### Step 2. Generation

To generate a set of preferences for leisure activities based on theoretical and empirical evidence that represented the conceptual area of the topic, we carried out three steps.

First, we conducted an evidence map with the aim of mapping the different instruments that assess preferences for everyday living (e.g., leisure, sexuality, eating and drinking) (Rommerskirch-Manietta et al., 2022). Then, based on the evidence map results, one researcher (M. Rommerskirch-Manietta) extracted the clearly stated items to assess preferences for leisure activities from the identified instruments. This resulted in a total of 361 theoretically generated items.

For the second step, we planned to involve a minimum of 10 people receiving ADS to ensure a variety of different opinions on the topic (Kane & Trochim, 2007).

Fifteen people receiving ADS voiced their interest in being involved in the empirical generation. One person could not be involved in this step due to an extended hospital stay. Face-to-face, semistructured interviews were conducted (M. Rommerskirch-Manietta) with them on site at the three ADS centers. Additionally, to empirically generating preferences for leisure activities, the aim of these interviews was to gain insight into the people’s understanding of the ADS center as a place for leisure. The interviews were audio recorded and length ranged from 18 to 75 min, with a mean length of 41 min. The interviews were analyzed via reflexive thematic analysis and led to the generation of 80 preferences for leisure activities (Rommerskirch-Manietta, Purwins, et al., 2023).

Finally, one researcher (M. Rommerskirch-Manietta) merged the theoretically and empirically generated items and excluded duplicates. Afterward, two researchers (M. Rommerskirch-Manietta, C. Manietta) reviewed the various items for redundancy (theoretically: Skip-Bo, Poker; empirically: playing cards), thus merging them (resulting in: playing cards) and discussed this with the last author (M. Roes). The aim of merging the items was to further reduce the number of items and make them manageable for the upcoming *structuring* step. To achieve this, the focus was on the empirically generated items, supplemented by the theoretical ones where appropriate, aiming to achieve saturation of possible preferences for leisure activities of people receiving ADS. The focus on the empirical items also followed the goal of honoring the “voice” of the people actively involved in the concept mapping. The voice of the persons involved was therefore not erased or devalued here; rather it was supplemented. This resulted in 80 different preferences for leisure activities, which mainly corresponded to those from the interviews (Table 2). Regarding similar concept mapping studies (Rosas & Ridings, 2017), the number of our generated items was in the same range and was consistent with Concept Systems’ recommendation regarding the manageability of the number of items in concept mapping studies (Concept Systems, 2023).

**Table 2.** Final Set of Generated Preferences for Leisure Activities

Preferences
1. Gardening
2. Distilling/brewing
3. Cooking
4. Baking
5. Singing
6. Playing instruments
7. Painting & drawing
8. Doing handcrafts
9. Doing mechanics
10. Reading
11. Listening to music
12. Watching TV
13. Volunteering
14. Mentoring
15. Initiating interest groups
16. Improving knowledge and skills
17. Learning new things
18. Reminiscing
19. Relaxing
20. Doing nothing
21. Taking time for myself
22. Wellness
23. Shopping
24. Going to restaurants
25. Going to parks
26. Sightseeing
27. Going to church
28. Attending sporting events
29. Visiting fun fairs
30. Going to cabarets
31. Attending concerts
32. Going to the opera
33. Attending festivals
34. Going on vacation
35. Playing ball games
36. Playing toss
37. Bowling
38. Playing mentally stimulating games
39. Swimming
40. Sailing
41. Roller skating
42. Dancing
43. Climbing
44. Doing gymnastics
45. Walking
46. Running
47. Cycling
48. Hand-picking
49. Dog walking
50. Dog sports
51. Caring for others
52. Being around other people
53. Talking with others
54. Getting to know other people
55. Building friendships
56. Playing pranks
57. Writing to pen pals
58. Being a club member
59. Spending time with family and friends
60. Spending time with my or other children
61. Spending time with animals
62. Playing board games
63. Doing quizzes
64. Solving riddles
65. Doing crossword puzzles
66. Playing memory games
67. Doing puzzles
68. Playing cards
69. Playing lottery games
70. Rolling dice
71. Driving car
72. Using public transportation
73. Trying out new things
74. Trying out everything
75. Celebrating your cultural festivals
76. Listening to cultural music
77. Doing or watching cultural sports
78. Explaining your own cultural practices to others
79. Enjoying your own cultural environment
80. Visiting your native country

#### Step 3. Structuring

For the preparation for sorting, one researcher (M. Rommerskirch-Manietta) and a research assistant searched Google for images that represented the 80 different preferences for leisure activities. To promote the involved peoples’ identification with the images, it was important for us to ensure that the preferences for leisure activities depicted in these images were performed by people who could represent people receiving ADS and were not taken in a residential care environment. Two to three images were selected for each preference, and their representativeness was discussed in two group meetings with the researchers (M. Rommerskirch-Manietta, C. Manietta) and the research assistant. As a result, the appropriate images were selected, printed on separate 30 × 21 cm cards, labeled in large font, and laminated.

Structuring of the various preferences was carried out on site at the three ADS centers. For this purpose, the different cards were randomly placed by a researcher (M. Rommerskirch-Manietta) either on a table or on the floor to ensure a quick overview of all 80 preferences for leisure activities for the people involved. Structuring was moderated by the researcher, performed by the people receiving ADS individually, and was divided into two steps, that is, sorting and rating.

**Figure 1.**
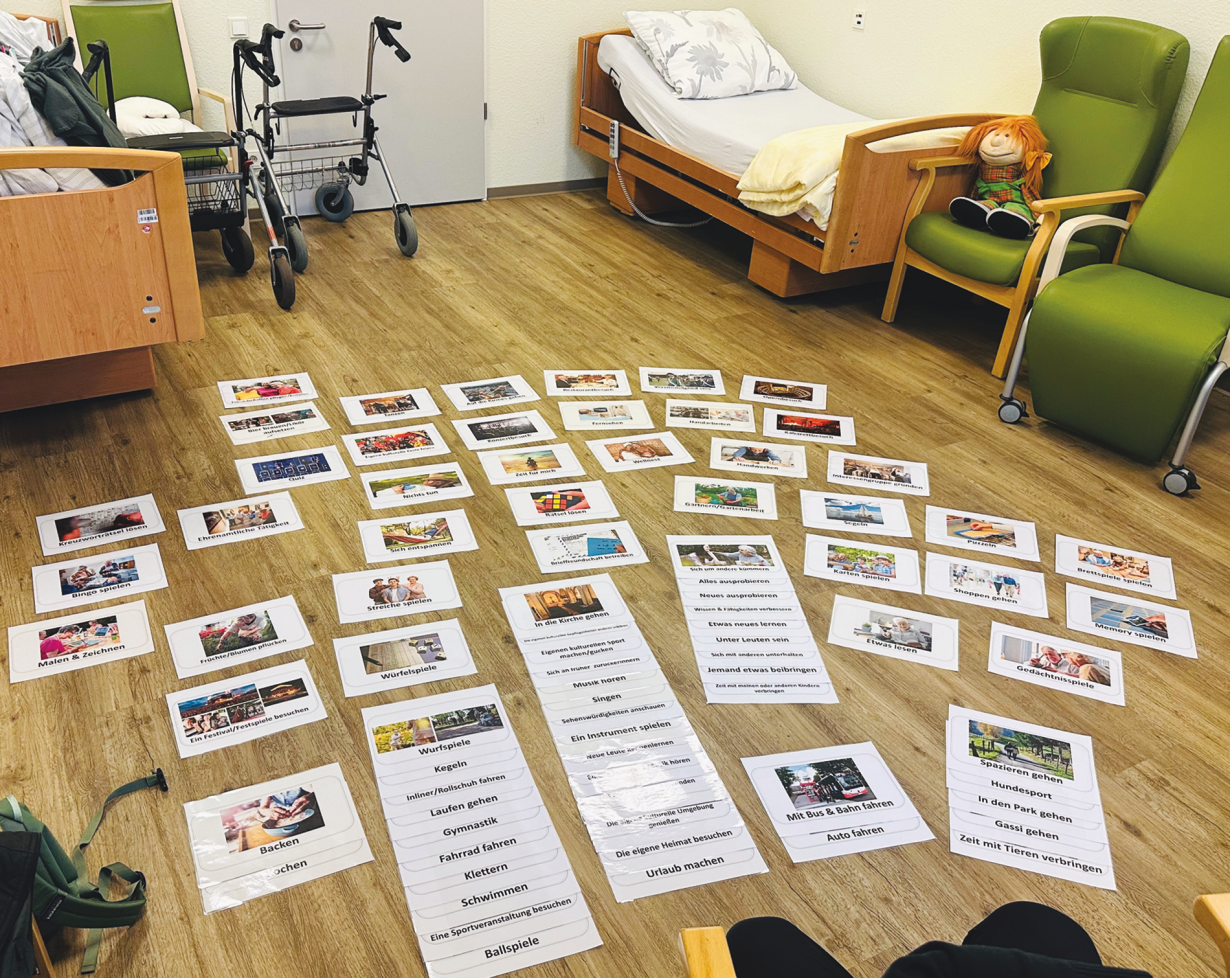
Sorting of the 80 preferences for leisure activities.

In the first step, each of the randomly placed cards was presented by the researcher to the person involved, followed by a narrative prompt to sort the different preferences for leisure activities into piles “in a way that makes sense for you” (Trochim & McLinden, 2017). Especially when the cards were placed on the floor, the researcher acted as a “helping hand” and performed the sorting based on the prompts from the people involved (Figure 1).

Second, all 80 preferences for leisure activities were rated by participants in terms of their importance for them on a large print Likert scale (ranging from *1—not important at all* to *4—very important*). The results of both steps were documented by the researcher via photographs and a results sheet.

Similar to the generation step, we planned to involve at least 10 people receiving ADS in the *structuring* step to provide a good working framework, ensure a variety of different opinions on the topic (Kane & Trochim, 2007), and to individually consider the possible support needs due to the forms of care needs, for example, symptoms of dementia, of the people involved (Lendon & Singh, 2021).

Overall, 12 of the 16 people receiving ADS voiced their interest in being involved in the *structuring* step. The other four people were either in a palliative care situation at home or had moved into a nursing home. Of these 12 people, 10 completed structuring, and the length of their involvement ranged from 30 to 120 min, with a mean length of 74 min. Reasons for not completing the structuring step included a lack of interest in sorting.

Finally, one researcher (M. Rommerskirch-Manietta) imported the results into the web-based Concept System Global Max software to archive, edit, and merge all sorting results in a binary square symmetric similarity matrix (Concept Systems, 2023).

#### Step 4. Representation

For the representation of our data (Trochim & McLinden, 2017), a researcher (J. M. Bergmann) exported the binary square symmetric similarity matrix and imported it into R statistical software version 4.2.1 for the statistical analyses and production of the graphical representation (R Core Team, 2022). The researcher used a combined application of multidimensional scaling (MDS), agglomerative hierarchical cluster (AHC) analysis (Trochim & McLinden, 2017) and descriptive statistics.

First, the researcher (J. M. Bergmann) performed three-dimensional ordinal MDS to transform the binary square symmetric similarity matrix into Euclidean distances and display them in a spatial configuration with the R package *smacof* using the *smacofSym* function (de Leeuw & Mair, 2009). Despite the usual use of two-dimensional ordinal MDS (Trochim & McLinden, 2017), we decided to perform three-dimensional MDS to increase the goodness of fit. We also considered a *secondary approach* for ties (“ties kept tied”); that is, equal proximities must be mapped into equal distances (Borg et al., 2012). This was done because of the number of sorts, items, and ties in sorts needed to present the best possible representation of the empirical data. Our calculated STRESS 1 value (Kruskal, 1978) for our three-dimensional solution was 0.226 (cutoff for a goodness of fit of 80 items of 0.297), signifying better internal representational validity than the higher stress value of 0.351 for the two-dimensional solution (cutoff for goodness of fit of 80 items of 0.388) (Rosas & Kane, 2012; Sturrock & Rocha, 2000).

Next, AHC analysis was performed with the R package *FactoMineR* using the *HCPC* function to identify clusters of items that are mapped in the geometric structures of the MDS (Lê et al., 2008). The aim was to find an appropriate cluster solution that minimized the variability of the “within cluster” or maximized the variability of the “between clusters.” This resulted in a statistical solution with six clusters that explained 72% of all variance. Based on the results, one researcher (M. Rommerskirch-Manietta) performed an accompanying interpretive analysis reviewing possible cluster solutions beginning from 15 to 5 clusters. This resulted in a solution with 14 clusters that appeared to be detailed and interpretable (Trochim & McLinden, 2017). The interpretive solution was discussed with one researcher (J. M. Bergmann) and compared to the statistical solution. As a result, a 12-cluster solution was chosen because it represented the best balance between the statistical and interpretive analysis results (89% of all variance, detail, and interpretability) (Kane & Trochim, 2007). According to the results, we produced a final graphical representation of the three-dimensional cube with 12 clusters and discussed this in the research group (M. Rommerskirch-Manietta, C. Manietta, J. M. Bergmann, M. Roes).

Finally, the ratings of the importance of the different preferences for leisure activities and clusters were analyzed using descriptive statistics.

#### Step 5. Interpretation

In preparation for the *interpretation* step, one researcher (M. Rommerskirch-Manietta) printed the graphic representation of the three-dimensional cube with 12 clusters on a 59 × 84 cm poster and organized the sorting cards with the images of the different preferences for leisure activities according to the 12 clusters. Additionally, a list of the results related to the importance ratings was prepared. All materials were intended to facilitate the interpretation of the conceptualization by the people receiving ADS (Trochim & McLinden, 2017).

We focused on working with the same people in the *interpretation* step as in the *generation* and the *structuring* steps because relationship building and thus mutual trust appeared to be an essential prerequisite for group discussion and the interpretation of the results (Waite et al., 2019). Because seven people from two of the three ADS centers with different attendance days expressed their interest in being involved in the interpretation, a joint meeting in one ADS center on 1 day was not possible. Consequently, we divided the seven people into three groups, with one group in one ADS center and two groups in another. Nevertheless, to ensure a collaborative result in this concept mapping step, we proceeded in two steps.

For the first step, we organized a discussion meeting for each group at the respective ADS center. At this meeting, one researcher (M. Rommerskirch-Manietta) first gave an introduction and overview of the 12 identified clusters with the different preferences for leisure activities and their importance ratings using the poster and the list of ratings. Subsequently, the researcher placed the different sorting cards sorted by the clusters at the respective distance between the clusters either on the table or on the floor. The people involved were then invited to discuss, interpret, and label the cluster. This discussion was moderated by the researcher, facilitated by prompts such as “What do the different preferences for leisure activities have in common” or “What do you associate with these preferences for leisure activities.” This step took an average of 115 min for each group and resulted in three different labels (one from each group) for each cluster (a total of 36 labels). Additionally, field notes were made by the researcher after each discussion meeting. After the discussion meetings, each cluster label was printed by the researcher in large font on separate 30 × 21 cm cards, which were laminated and assigned to the corresponding sorting cards.

For the second step, a voting meeting was scheduled with the three groups at their respective ADS center. This meeting was moderated by the same researcher (M. Rommerskirch-Manietta), and the different laminated label cards with the corresponding sorting cards were presented to the people involved. After the overview and a discussion, each group member voted for their preferred label for each cluster. The length of this voting process lasted an average of 74 min per group.

The ratings were analyzed by a researcher (M. Rommerskirch-Manietta) and discussed in the research group in the context of the field notes, and the label with the most votes for each cluster was chosen.

### Trustworthiness

To ensure the trustworthiness of our results, we involved people receiving ADS in the *generation*, *structuring*, and *interpretation* steps of our concept mapping. By involving them in these different steps, we were able to ensure that people receiving ADS mentioned and clustered their preferred leisure activities according to their life experience (accuracy) while reflecting on and labeling the clustering, which led to a thick description of our results (credibility). By honoring their “voice,” the research question was answered in their terms (coherence). Furthermore, this approach supports the transferability and trustworthiness of our study results (Rosas & Ridings, 2017; Yadav, 2021).

## Results

**Figure 2.**
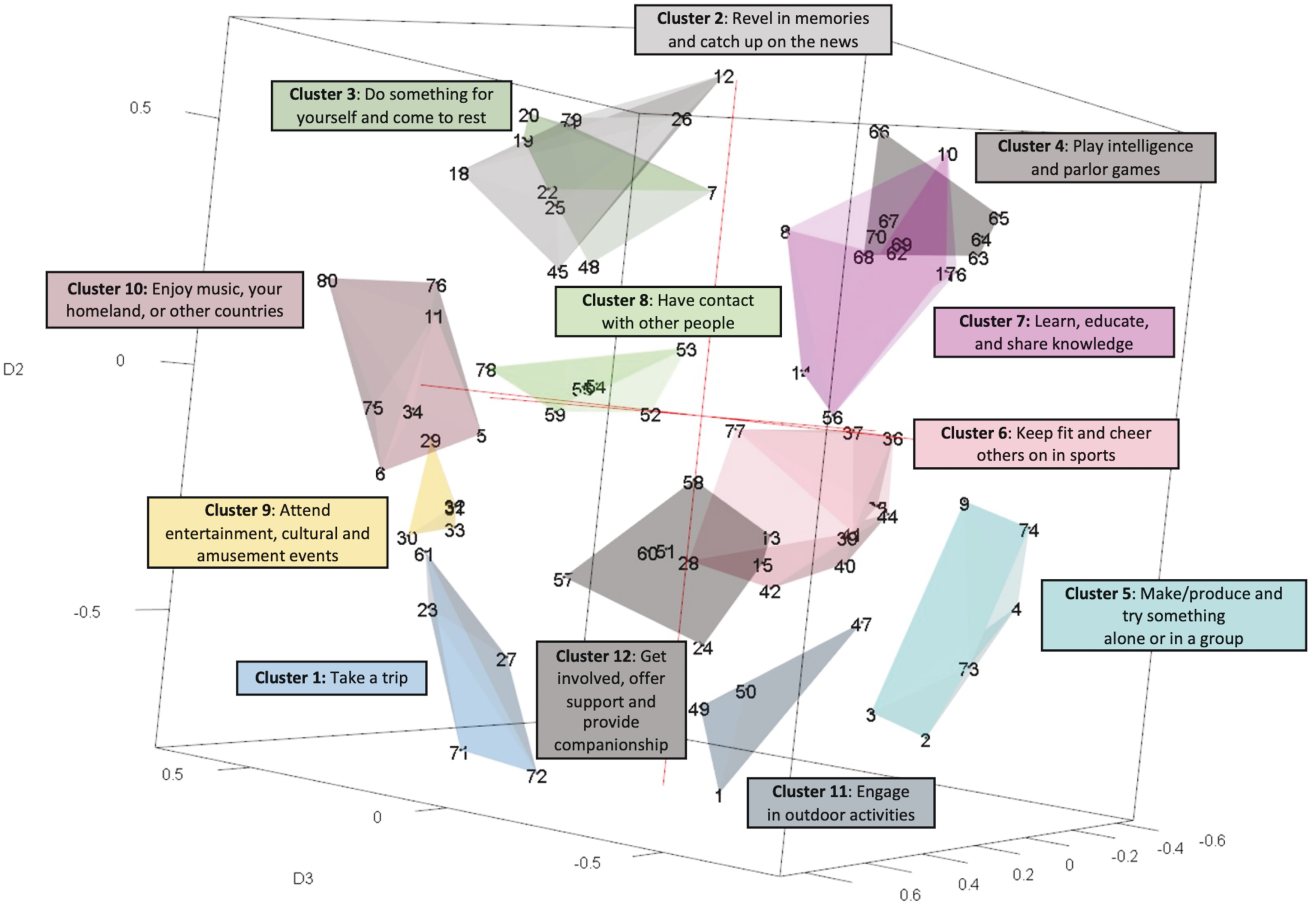
Three-dimensional cube with 12 clusters.

Our final conceptualization of preferences for leisure activities of people receiving ADS featured 12 clusters (Figure 2). The clusters were labeled by the people receiving ADS as follows: 1. *Take a trip*, 2. *Revel in memories and catch up on the news*, 3. *Do something for yourself and come to rest*, 4. *Play intelligence and parlor games*, 5. *Make/produce and try something alone or in a group*, 6. *Keep fit and cheer others on in sports*, 7. *Learn, educate, and share knowledge*, 8. *Have contact with other people*, 9. *Attend entertainment, cultural, and amusement events*, 10. *Enjoy music, your homeland, or other countries*, 11. *Engage in outdoor activities*, and 12. *Get involved, offer support, and provide companionship*. A detailed overview of the different preferences for leisure activities of people receiving ADS, sorted into the 12 clusters, is provided in Table 3.

**Table 3.** Importance Rating for the Preferences for Leisure Activities and Clusters

Preferences	Importance rating *Mean*^a^ (SD)
**Cluster 2:** Revel in memories and catch up on the news	***3.42* (0.29)**
25. Going to parks	*3.80* (0.42)
45. Walking	*3.70* (0.48)
12. Watching TV	*3.40* (1.07)
18. Reminiscing	*3.40* (0.69)
26. Sightseeing	*3.10* (1.10)
79. Enjoying your own cultural environment	*3.10* (1.28)
**Cluster 8:** Have contact with other people	***3.18* (0.66)**
59. Spending time with family and friends	*3.90* (0.31)
52. Being around other people	*3.60* (0.69)
53. Talking with others	*3.50* (0.70)
55. Building friendships	*3.10* (1.10)
54. Getting to know other people	*3.00* (0.81)
78. Explaining your own cultural practices to others	*2.00* (1.24)
**Cluster 10:** Enjoy music, your homeland, or other countries	***2.97* (0.58)**
11. Listening to music	*3.90* (0.31)
75. Celebrating your cultural festivals	*3.30* (0.94)
34. Going on vacation	*3.20* (1.22)
80. Visiting your native country	*2.90* (1.44)
76. Listening to cultural music	*2.80* (1.39)
5. Singing	*2.70* (1.49)
6. Playing instruments	*2.00* (1.24)
**Cluster 7:** Learn, educate, and share knowledge	***2.82* (0.65)**
10. Reading	*3.40* (1.07)
16. Improving knowledge and skills	*3.20* (1.03)
17. Learning new things	*3.10* (0.99)
8. Doing handcrafts	*3.00* (1.33)
14. Mentoring	*2.60* (1.42)
56. Playing pranks	*1.60* (0.84)
**Cluster 3:** Do something for yourself and come to rest	***2.75* (0.82)**
19. Relaxing	*3.60* (0.51)
21. Taking time for myself	*3.50* (0.70)
20. Doing nothing	*3.40* (0.96)
48. Hand-picking	*2.10* (1.19)
7. Painting & drawing	*2.00* (1.33)
22. Wellness	*1.90* (1.20)
**Cluster 5:** Make/produce and try something alone or in a group	***2.70* (0.61)**
73. Trying out new things	*3.30* (1.05)
3. Cooking	*3.00* (1.05)
4. Baking	*3.00* (1.24)
74. Trying out everything	*2.80* (1.13)
9. Doing mechanics	*2.30* (1.33)
2. Distilling/brewing	*1.60* (1.07)
**Cluster 4:** Play intelligence and parlor games	***2.63* (0.29)**
62. Playing board games	*2.90* (1.37)
64. Solving riddles	*2.80* (1.13)
65. Doing crossword puzzles	*2.80* (1.22)
70. Rolling dice	*2.80* (1.31)
38. Playing mentally stimulating games	*2.70* (1.41)
67. Doing puzzles	*2.70* (1.15)
69. Playing lottery games	*2.70* (1.33)
68. Playing cards	*2.60* (1.26)
66. Playing memory games	*2.40* (1.50)
63. Doing quizzes	*1.90* (1.28)
**Cluster 1:** Take a trip	***2.50* (0.21)**
27. Going to church	*2.70* (1.25)
61. Spending time with animals	*2.70* (1.25)
72. Using public transportation	*2.50* (1.08)
23. Shopping	*2.40* (1.34)
71. Driving car	*2.20* (1.39)
**Cluster 12:** Get involved, offer support, and provide companionship	***2.46* (0.82)**
60. Spending time with my or other children	*3.80* (0.62)
51. Caring for others	*3.20* (1.13)
24. Going to restaurants	*2.80* (1.31)
58. Being a club member	*2.10* (1.44)
13. Volunteering	*1.90* (0.87)
15. Initiating interest groups	*1.70 (0.94)*
57. Writing to pen pals	*1.70* (1.05)
**Cluster 11:** Engage in outdoor activities	***2.30* (0.50)**
47. Cycling	*2.80* (1.13)
49. Dog walking	*2.50* (1.35)
1. Gardening	*2.30* (1.15)
50. Dog sports	*1.60* (1.07)
**Cluster 9:** Attend at entertainment, cultural, and amusement events	***2.16* (0.44)**
31. Attending concerts	*2.70* (1.49)
29. Visiting fun fairs	*2.50* (1.35)
33. Attending festivals	*2.10* (1.44)
30. Going to cabarets	*1.90* (1.44)
32. Going to the opera	*1.60* (0.96)
**Cluster 6:** Keep fit and cheer others on in sports	***2.10* (0.60)**
39. Swimming	*3.10* (1.19)
37. Bowling	*2.90* (1.19)
77. Doing or watching cultural sports	*2.60* (1.26)
36. Playing tossing	*2.30* (1.25)
44. Doing gymnastics	*2.30* (1.25)
35. Playing ball games	*2.20* (1.39)
28. Attending sporting events	*2.10* (1.44)
42. Dancing	*2.10* (1.44)
43. Climbing	*1.50* (0.84)
46. Running	*1.50* (0.84)
40. Sailing	*1.30* (0.94)
41. Roller skating	*1.30* (0.94)

*Notes*: SD = standard deviation.

^a^Ranges from 1.0 to 4.0; a higher mean indicates higher importance.

Regarding the rating of the importance (Table 3) (ranging from *1—not important at all* to *4—very important*) of the different preferences for leisure activities, the people receiving ADS rated the preferences for leisure activities in the second cluster *Revel in memories and catch up on the news [3.42 M 0.29 SD]*, including the items *Going to parks [3.80 M 0.42 SD]*, *Walking [3.70 M 0.48 SD]*, *Watching TV [3.40 M 1.07 SD]*, *Reminiscing [3.40 M 0.69 SD]*, *Sightseeing [3.10 M [1.10 SD]*, and *Enjoying your own cultural environment [3.10 M 1.28 SD]* as so important for them that this cluster had the highest importance value among all the clusters.

In contrast, cluster six *Keep fit and cheer others on in sports [2.10 M 0.60 SD]*, included the most preferences for leisure activities that were rated as less important by the people receiving ADS. This cluster consisted of the following: *Swimming [3.10 M 1.19 SD]*, *Bowling [2.90 M 1.19 SD]*, *Doing or watching cultural sports [2.60 M 1.26 SD]*, *Playing toss [2.30 M 1.25 SD]*, *Doing gymnastics [2.30 M 1.25 SD]*, *Playing ball games [2.20 M 1.39 SD]*, *Attending sporting events [2.10 M SD 1.44]*, *Dancing [2.10 M 1.44 SD]*, *Climbing [1.50 M 0.84 SD]*, *Running [1.50 M 0.84]*, *Sailing [1.30 M 0.94 SD]*, and *Roller skating [1.30 M 0.94 SD].*

Overall, all 12 clusters included at least two preferences for leisure activities that were rated as important by the people involved *(cutoff > 2.50 M)*. Additionally, the top three most/least importantly rated preferences for leisure activities across all clusters were *Spending time with family and friends (3.90 M 0.31 SD)/Roller skating (1.30 M 0.94 SD)*, *Listening to music (3.90 M 0.31 SD)/Sailing (1.30 M 0.94 SD)*, and *Going to parks (3.80 M 0.42 SD)*/*Running (1.50 M 0.84 SD).*

## Discussion and Implications

To our knowledge, our study was the first to explore for the content and structure of preferences for leisure activities of people receiving ADS. Our results imply that people receiving ADS have diverse preferences for leisure activities, including social, learning, productive, resting, play, travel, and physical activities. We identified a 12-cluster concept of preferences for leisure activities of people receiving ADS, with all clusters including at least two important preferences. This result indicated the importance of each cluster of our conceptualization and thus validated the diversity of the concept for the people involved. This diversity is in contrast to the preferences assessed thus far with instruments for other populations or care environments (Rommerskirch-Manietta et al., 2022) and seems more linkable to the self-reported outcomes of leisure (well-being, hedonism, social connectedness, identity (re)construction, and learning) of older people living in the community (de Araujo & da Rocha, 2019).

With a closer look at the structures and labels of the most important and least important clusters (most important: *Revel in memories and catch up on the news*/least important: *Keep fit and cheer others on in sports*), it is notable that the most important cluster includes aspects of identity and foreignness, passivity and activity and was therefore likely labeled by the people involved with two opposite poles: *revel in memories* and *catch up on the new*s. Based on the cluster’s label, the preferences *Reminiscing* (Revel in memories) and *Watching TV (Catch up on the news)* appear to be the starting point for the labeling that connects the other included preferences in this cluster and allows us to assign them to the two poles. The common features of the clustered items expressed in the cluster labels seem particularly interesting for the planning of a preference-based leisure activity program in ADS centers, as it allows healthcare professionals in ADS centers to provide a *complex* preference-based leisure activity intervention that incorporates a bundling of different assessed importance preferences (e.g., walking to familiar places in the neighborhood, sharing similar stories experienced at the location, and receiving information about new developments in the local area), likely addressing more than one of the abovementioned outcomes (e.g., well-being, identity (re)construction) (Skivington et al., 2021). Consequently, both the cost and staffing required to implement a preference-based leisure program remain low for ADS center providers, which is an important influencing factor for its feasibility and implementability (Damschroder et al., 2022).

Furthermore, notably, our least important cluster (*Keep fit and cheer others on in sports*) included mostly preferences for different types of physical activity. One explanation for the least importance of these preferences could be related to the barriers that older people face in participating in physical leisure activities. Accordingly, studies report that participation in physical leisure activities often declines or is completely discontinued by older people, for example, due to health conditions, fear of falling and/or being injured, or their social environment (Behrens et al., 2020; Moschny et al., 2011; Webster et al., 2022). Consequently, the importance of these preferences may diminish for them, as they no longer see a way to fulfill their preferences (Curyto et al., 2016). However, knowledge of preferences and the linked barriers could lead to interventions by healthcare professionals in ADS centers to address these barriers (e.g., lowering the intensity or duration time and providing company or support). This could lead to an active (re)engagement of people receiving ADS in their preferences, for example, physical leisure activities (Heid et al., 2022) or, if barriers cannot be addressed, to creative consideration with the people receiving ADS whether adapting the preference (e.g., dancing → training others in dancing) is a way to fulfill this preference and satisfy them.

Exploring possibilities of the utilization of our results, our cluster labels, and the consideration of barriers for preferences for leisure activities seem to be key aspects for the development of an instrument to assess preferences for leisure activities of people receiving ADS.

First, our cluster labels appear to indicate key potential questions that could be adapted and adopted for an instrument to assess the preferences of people receiving ADS. The structure of the Preference for Everyday Living Inventory (PELI) could provide guidance here. In the first step, the PELI is used to assess the importance of the respective item by the interviewee (ranging from *very important* to *not important at all*), followed by a more detailed open-ended or dichotomized question to specify the preferences (Van Haitsma et al., 2013). Consequently, the labels of our clusters could be adapted into questions that are rated in the first step based on importance; if rated as important, the second step for the following questions is triggered to specify the preferences for leisure activities based on our cluster items. Considering such a two-step structure would likely result in an instrument with a reduced number of main questions (*n* < 20), which seems feasible for care providers (Abbott et al., 2021) and may prevent interviewee fatigue.

Second, addressing the aspect of barriers seems to be an essential point to support the (re)engagement in preferences for, for example, physical leisure activities of people receiving ADS. Again, the PELI could provide guidance here. The PELI uses the answer option “Important, but can’t do” in addition to the options related to the importance of identifying preferences that require support to fulfill due to barriers (Curyto et al., 2016). Adopting this answer option appears to be essential in the context of assessing the leisure activity preferences of people receiving ADS to assess the importance of a preference that is as uninfluenced by barriers as possible.

### Limitations

Our concept mapping has some limitations. Our sample is homogeneous in terms of their cultural diversity. Three people with a migration background participated in our concept mapping. Especially regarding cultural-specific preferences, including more people from different cultural backgrounds could have broadened our results. However, due to a lack of statistical data and studies in Germany, it is unknown how diverse the population of people receiving ADS is in Germany and whether our sample reflects this population. Studies report that people with a migration background (e.g., people from Turkey) often do not use professional care services in Germany (Tezcan-Güntekin et al., 2022), which is reflected in our study sample.

Moreover, it needs to be considered that during our interviews focusing on the identification of preferences for leisure activities, *socially desirable* answers may have been given by the people receiving ADS. As shown in a study by Katz (2000), older people perceive a *busy body* as an expectation of *society* for their age group. This may be reinforced, particularly in care communities (e.g., ADS centers), by healthcare professionals keeping them active and a collective morality shared among the older people about being active (e.g., self-disciplining). This may have encouraged responses related to active rather than passive activities in our interviews. Nevertheless, preferences such as *doing nothing* or *relaxing* were also mentioned by the people involved in our interviews. This highlights the importance of preference-based services because, according to Van Haitsma et al. (2020), honoring preferences can be understood as a mechanism through which someone chooses to engage in an activity or not. Additionally, it indicates that preference-based leisure activity services are a prerequisite for the transformation of ADS centers to lively communities based on a person-centered philosophy of care.

Furthermore, concept mapping seems to be a structured but complex research approach when involving people from the public in research. This complexity and the associated challenge in the required high level of abstraction led us to supplement the various concept mapping steps with individual activities and methods (e.g., relationship building, promoting autonomy and safety, using sorting cards) to support the people involved so that the steps remained feasible for them. However, the required abstractness of the research approach led us to adapt our procedure so that we did not identify additional higher areas of meaning in our conceptualization by the people involved. Therefore, in view of the increase in research involving the public (Griffith et al., 2023; Rommerskirch-Manietta, Manietta, et al., 2023), it seems necessary to further develop research methods so that they are more feasible for the nonacademic population.

## Conclusion

Our conceptualization of preferences for leisure activities of people receiving ADS opens the field for further research. In this context, further development (e.g., by conducting cognitive interviews) (Willis, 2005) with a larger sample of people receiving ADS, psychometric testing, and implementation of a feasible instrument to assess the preferences for leisure activities of people receiving ADS seems a key opportunity that could provide the possibility for full-scale data collection leading to further insights into these preferences and outcome evaluations. Moreover, our study reported transparent, transferrable methodological knowledge regarding the involvement of people receiving ADS in concept mapping. Thus, further studies can continue our work in verifying the 12-cluster conceptualization in an international and culturally more diverse environment, which may help to advance the theory building on preferences for leisure activities of people receiving ADS. Furthermore, it seems important to investigate to what extent barriers (e.g., decline in physical functioning, stigma, self-ageism) to preferences for leisure activities (e.g., preferences in the cluster *keep fit and cheer others on in sports*) influenced the importance ratings of the people involved in our concept mapping. If these were only rated as, for example, *not important at all* due to experienced barriers, further research needs to focus on interventions to face these barriers (Duan et al., 2023). Finally, addressing the abovementioned further research aspects will advance this *revealed* field of research and may provide guidance for researchers, practitioners, and providers in assessing preferences and developing complex preference-based interventions, as well as implementing a preference-based leisure program in ADS centers and evaluating people’s outcomes.

## Data Availability

There was no preregistration for this study. Data are available on request from the author.
